# Celebrating the third World Field Epidemiology Day: a focus on MediPIET the field epidemiology training programme in Mediterranean and Black Sea countries

**DOI:** 10.2807/1560-7917.ES.2023.28.36.2300474

**Published:** 2023-09-07

**Authors:** Aleksandar Obradovic

**Affiliations:** 1 Institute for Public Health of Montenegro, Podgorica, Montenegro

**Keywords:** Field epidemiology, World Field Epidemiology day, WFED, training programme, capacity building, workforce capacity, FETP, MediPIET

On 7 September 2023, we celebrate the third World Field Epidemiology Day (WFED). The WFED initiative was launched in 2021 by the Training Programs in Epidemiology and Public Health Interventions Network (TEPHINET), and along with more than 60 institutions or organisations, *Eurosurveillance* has supported this initiative from the start [1,2].

Important goals of field epidemiology are to anticipate, prevent, detect, and deal with potential and existing sources of harm and/or threats to public health. In some way, field epidemiologists are health guardians, who need to be well prepared to fulfil their tasks. One year ago, to mark the WFED 2022, an editorial in this journal highlighted the contribution of epidemiologists to strengthening health systems’ preparedness and response to public health threats and the resulting need to recruit and maintain a well-trained and competent field epidemiology workforce. The editorial further pointed out how fellows of the European Centre for Disease Prevention and Control (ECDC) Fellowship Programme, both in the Field Epidemiology path (EPIET) and the Public Health Microbiology path (EUPHEM), have for example contributed to the COVID-19 pandemic response [2].

Worldwide, the Field Epidemiology Training Programs (FETP) network, has grown constantly and starting from the year 2013, the Mediterranean and Black Sea Programme for Intervention Epidemiology Training (MediPIET) has been operational as an initiative of the European Commission to enhance health security in this region [3]. Covering a large geographical region and comprising 21 countries on three continents (Europe, Africa and Asia) with different cultures, yet facing similar public health challenges, the network has some unique features. Up to mid-2021, MediPIET was co-ordinated by several different international public health authorities and institutions and since then it became part of the European Union (EU) Initiative on Health Security [3]. In spite of the initially challenging years, MediPIET was an important element of capacity-building for every member country of the network.

First of all, it initiated and helped preserving an FETP ‘flame’ in participant countries, and hereafter, formalised or non-formalised cascading activities were conducted. Some of those cascading activities became fully functional national FETPs in several countries. For example, MediPIET alumni had an important role in the development and leading of new training programmes in Georgia, Lebanon and Tunisia, and also continue to contribute to programmes as faculty. MediPIET also inspired activities of continuing medical education. For example, in Montenegro in 2017 and 2018, almost all senior epidemiologists from local epidemiological services, and the majority of residents in Epidemiology at that moment participated in a few adjusted and translated MediPIET modules. Those activities exemplify the multiplication effect of the programme itself.

Throughout the years, MediPIET became more tightly connected with other FETPs, especially with the ECDC Fellowship Programme, and in 2022 it also became a member of TEPHINET. In their interactions, the different programmes are experiencing various types of benefits. On the one hand, for MediPIET countries, the connection with EPIET/EUPHEM is providing new and more opportunities to empower fellows' knowledge. On the other hand, for countries participating in the ECDC Fellowship Programme there are prospects to establish a broader and more powerful network, and to learn about and experience challenges of neighbouring countries. This is especially important considering that communicable diseases do not stop at borders and different state settlements, and threats elsewhere may also become our own threats.

Any major infectious disease event or crisis impacting on public health and society provides greater visibility of field epidemiology. The COVID-19 pandemic which lasted for over 2 years, provided an opportunity to test our current capacities to deal with challenges posed by a newly-introduced respiratory virus, as well as to advocate for the importance of field epidemiology and the importance of its continuous improvement. Hence, this is the right moment to try to establish even stronger networks without leaving anyone behind. We can only prevent and control communicable diseases if we are, at country levels, equally capable to handle them. In parallel with the COVID pandemic, we witnessed the beginning of a new technological revolution with the advent of a broader use of artificial intelligence and an accelerated global digitalisation process. We thus need to work harder on connecting new IT solutions and their possibilities with field epidemiology to help us improve our surveillance systems.

An important challenge in the Mediterranean and Black Sea region continues to be the lack of well-trained field epidemiologists which makes the role of MediPIET in participating countries important in the mission to achieve enough adequately trained workforce to be able to work cooperatively to tackle cross-border health security threats.

In this issue of *Eurosurveillance*, two Perspectives focus on field epidemiology to tie in with WFED. One Perspective by Hahné et al. presents results from a study and discussion within the EPIET alumni network (EAN) conducted among EPIET and EUPHEM alumni and current fellows, about the evolution of the profession of field epidemiology and about the perception of current, and the anticipation of future challenges and opportunities emerging with a view of the near future [4]. These recent results will provide a basis to look back to and see what the main expectations of field epidemiologists were at the beginning of the 2020s and in how far these have changed over time. Another Perspective by Schaeffer et al. is also connected to the EAN [5]. It reflects findings of a survey conducted among EAN members about satisfaction of the alumni with skills and competencies gained through fellowship and the importance of cross-border collaboration. Respondents deemed that they had learned new skills and competencies which contributed to an improved performance in their professional role compared to before the fellowship.

Improving competencies and strengthening capacities are at the core of the FETPs’ mission and on WFED we should also remember an important foundation of field epidemiology – not to forget the past and to learn from previous experiences! We need to remember and to continue improving ourselves, to continue improving field epidemiology. Only by preserving the memory of important lessons and learning from these we can achieve the necessary continuity in the process of becoming better prepared for future public health crises.

## Note

Aleksandar Obradovic is the co-chair of the MediPIET Training Centers Forum (TCF), and the National focal point of MediPIET in Montenegro.
